# A Review: PI3K/AKT/mTOR Signaling Pathway and Its Regulated Eukaryotic Translation Initiation Factors May Be a Potential Therapeutic Target in Esophageal Squamous Cell Carcinoma

**DOI:** 10.3389/fonc.2022.817916

**Published:** 2022-04-28

**Authors:** Ran Huang, Qiong Dai, Ruixue Yang, Yi Duan, Qi Zhao, Johannes Haybaeck, Zhihui Yang

**Affiliations:** ^1^ Department of Pathology, The Affiliated Hospital of Southwest Medical University, Luzhou, China; ^2^ Department of Human Anatomy, School of Basic Medical Sciences, Southwest Medical University, Luzhou, China; ^3^ Department of Cardiology, The Affiliated Hospital of Southwest Medical University, Luzhou, China; ^4^ Institute of Pathology, Neuropathology and Molecular Pathology, Medical University of Innsbruck, Innsbruck, Austria; ^5^ Diagnostic & Research Center for Molecular BioMedicine, Institute of Pathology, Medical University of Graz, Graz, Austria

**Keywords:** esophageal squamous cell carcinoma (ESCC), PI3K/AKT/mTOR signaling pathway, inhibitors, eukaryotic translation initiation factors (eIFs), therapeutic target

## Abstract

Esophageal squamous cell carcinoma (ESCC) is a malignant tumor developing from the esophageal squamous epithelium, and is the most common histological subtype of esophageal cancer (EC). EC ranks 10th in morbidity and sixth in mortality worldwide. The morbidity and mortality rates in China are both higher than the world average. Current treatments of ESCC are surgical treatment, radiotherapy, and chemotherapy. Neoadjuvant chemoradiotherapy plus surgical resection is recommended for advanced patients. However, it does not work in the significant promotion of overall survival (OS) after such therapy. Research on targeted therapy in ESCC mainly focus on EGFR and PD-1, but neither of the targeted drugs can significantly improve the 3-year and 5-year survival rates of disease. Phosphatidylinositol 3-kinase (PI3K)/protein kinase B (AKT)/mammalian target of rapamycin (mTOR) pathway is an important survival pathway in tumor cells, associated with its aggressive growth and malignant progression. Specifically, proliferation, apoptosis, autophagy, and so on. Related genetic alterations of this pathway have been investigated in ESCC, such as *PI3K*, *AKT* and *mTOR-rpS6K*. Therefore, the PI3K/AKT/mTOR pathway seems to have the capability to serve as research hotspot in the future. Currently, various inhibitors are being tested in cells, animals, and clinical trials, which targeting at different parts of this pathway. In this work, we reviewed the research progress on the PI3K/AKT/mTOR pathway how to influence biological behaviors in ESCC, and discussed the interaction between signals downstream of this pathway, especially eukaryotic translation initiation factors (eIFs) and the development and progression of ESCC, to provide reference for the identification of new therapeutic targets in ESCC.

## Introduction

### The General Status of Esophageal Carcinoma

In 2020, the incidence and mortality of esophageal carcinoma (EC) ranked tenth and sixth in the world, with about 70% of the cases affection males. According to statistics, in 2020, 544,076 people died due to EC worldwide1]. The incidence in eastern Asia is the highest in the world (1). EC mainly includes ESCC and esophageal adenocarcinoma (EAC). The incidence of ESCC is higher than EAC in Asia (2). Currently, the incidence of ESCC has decreased significantly in Asia (e.g., in China), probably due to the decline in poverty (3), but the mortality rate is still not optimistic.

With the improvement of various examination methods, the detection rate of esophageal squamous dysplasia is increasing, especially in the areas with high incidence of ESCC. Esophageal squamous dysplasia has the potential to develop into ESCC. The two can be regarded as a continuous pathological process (4). The symptoms of EC are very insidious. Early EC is often detected by gastroscopy, CT, or MRI (5). These examinations may reveal ulceration or protuberance on the mucosal esophageal surface, or thickening of the esophageal wall (5). As the lesion progresses, patients might have difficulty swallowing. They can only take half liquid diet or even liquid diet, until they are completely unable to eat and thus significantly lose weight. Some patients even feel chest pain (6).

**Figure 1:**
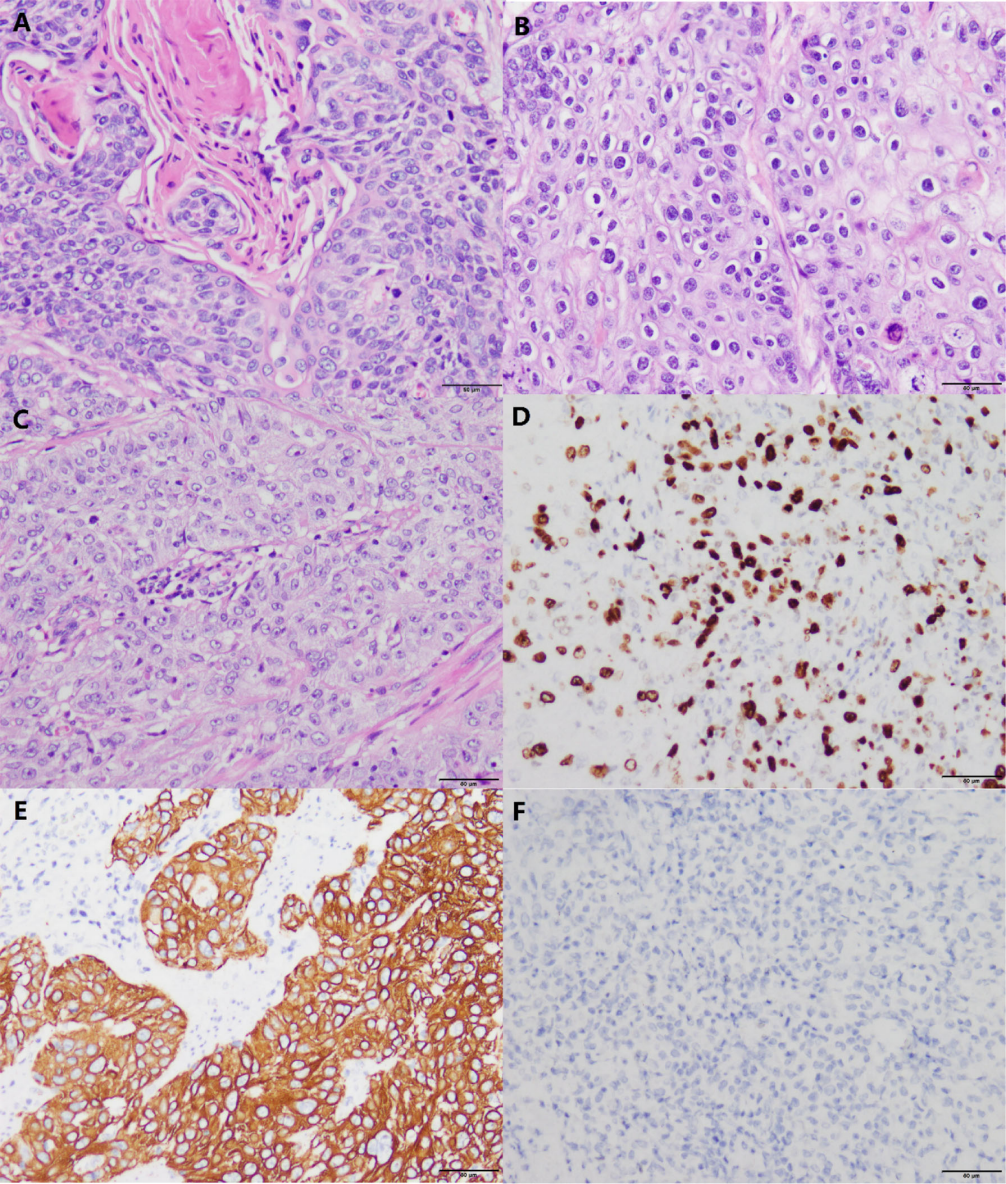
Histomorphology and immunohistochemistry in variably differentiated ESCC (own images, have not been published in elsewhere, the scale bar is 50 µm). **(A)** Highly differentiated ESCC (H&E stain, ×200). **(B)** Moderately differentiated ESCC (H&E stain, ×200). **(C)** Poorly differentiated ESCC (H&E stain, × 200). **(D)** In this case of poorly differentiated ESCC, the positive index of Ki-67 reaches 50% (Envision stain, ×200). **(E)** Poorly differentiated ESCC cells are positive for CK5/6 (Envision stain, ×200). **(F)** Poorly differentiated ESCC cells are negative for CK7 (Envision stain, ×200).

According to the degree of differentiation of ESCC, three grades can be differentiated: highly, moderately, and poorly (7) (**Figures 1A–C**). Many keratins and intercellular bridges can be seen in a highly differentiated ESCC. The poorly differentiated ESCC does not have keratin pearls and intercellular bridges. These cells are disorderly arranged hierarchy, with higher cellular atypia and nuclear pleomorphism.

Poorly differentiated ESCC and EAC are difficult to distinguish by HE staining alone (7). Immunohistochemistry (IHC) can distinguish them. In poorly differentiated ESCC, markers indicating squamous epithelial differentiation (e.g., CK5/6) (**Figures 1D, E**) are positive and markers indicating glandular epithelial differentiation are negative (e.g., CK7, CK20) (7) (**Figure 1F**). P63 is an indicator that has been shown to be positively expressed in ESCC (8). Some patients can have components of both squamous cell carcinoma and adenocarcinoma, which is termed adenosquamous carcinoma (9).

### The Methods and Efficacy of Current Treatments for ESCC

Most ESCC patients are diagnosed at advanced stage. Thus, more attention should be paid to prevention and early diagnosis. If this disease is diagnosed early, endoscopic therapy is possible. This can save the patient’s organs and improve the well-being (10, 11). Some scholars suggest that endoscopic ultrasound (EUS) should be performed before treatment to accurately evaluate the condition (it is not recommended for some patients with extreme esophageal stenosis) and guide the therapy. T staging and regional lymph node status are important prognostic factors for ESCC. EUS is a test comparable to PET, accurate for T staging and inexpensive. More importantly, EUS is superior to CT and MRI in the detection of lymph node involvement, with higher sensitivity (10).

In a randomized controlled trial conducted by Joel Shapiro and colleagues, the addition of neoadjuvant chemoradiotherapy (preoperative chemoradiotherapy) during surgery for patients with resectable EC was found to have an overall survival benefit (12). It was confirmed during the follow-up for a long term. In a phase III clinical trial conducted by Chinese scholars, it was also found that neoadjuvant chemoradiotherapy plus surgery (NCT01216527) improved the survival rate of locally advanced ESCC patients compared with surgery alone, with acceptable and controllable adverse events (13).

Currently, neoadjuvant chemoradiotherapy and esophagectomy are the mainly therapy for ESCC. In a guide to the management of EC, it is suggested that patients with locally advanced ESCC should receive neoadjuvant chemoradiotherapy (14). Multimodal therapy has advantages over performing surgical resection alone. Similarly, patients without metastatic disease should receive esophageal resection after neoadjuvant therapy, if the evaluation of surgery is safe (14).

Japanese scholar Masayuki Watanabe and colleagues found that patients with ESCC received neoadjuvant chemoradiotherapy plus surgical resection, the 3-year survival rate was 29.8% and the 5-year survival rate was 15.0% (15). This data indicates that there is still a large proportion of patients who do not achieve better outcomes. Researchers are still exploring other ways to treat ESCC.

Currently, many researchers are focusing on the treatment of ESCC by molecular targeting. EGFR and PD1 are the hottest targets. It has been demonstrated that EGFR is one of the cancer genes responsible for the common somatic copy number variations (SCNV) in ESCC (16). At the same time, some researchers found abnormally high expression of EGFR in ESCC (17). In a phase II, single-group, multicenter trial conducted in several Chinese hospitals, researchers found that icotinib (an EGFR tyrosine kinase inhibitor, TKI, NCT01855854) exhibited promising activity in advanced ESCC patients whose EGFR was overexpressed or amplified (18). These trial results showed that only a small number of patients respond well to icotinib. So, it can be speculated that ESCC patients may have developed resistance to EGFR-targeted therapy (18). Curtis R Chong and colleagues proposed that the resisting to EGFR-targeted therapy in tumor cells could be relevant to the abnormally activating of PI3K/AKT/mTOR pathway (19).

In the phase III AIO/EORTC clinical trial conducted by M. Moehler and colleagues, they found that the use of panitumumab (an anti-EGFR antibody) combined with cisplatin and 5-fluorouracil did not improve survival compared to unselected advanced ESCC patients who received 5-fluorouracil alone (20). This result supports further studies of serum and tumor biomarkers (20).

Programmed cell death protein 1 (PD-1) is an inhibitory receptor expressed on activated lymphocytes, can connect with the ligands of PD-L1 and PD-L2. And they are favorable to regulating the balance of T cell activation, immune tolerance, and immune-mediated tissue damage (21, 22). Blockading the immune checkpoint has fundamentally improved the treatment of melanoma patients (23). At the same time, many researchers are exploring its efficacy in other cancers (24). A monoclonal antibody targeting PD-1, Nivolumab, could increase tumor antigen-specific T cell proliferation and cytokine secretion *in vitro (* 25, 26). It has been approved for the treatment of many other diseases, for example, advanced non-small cell lung cancer and Hodgkin’s lymphoma (27, 28). Nivolumab has been approved for ESCC patients who have progressed after chemotherapy in Japan since February 2020 (29). Toshihiro Kudo and colleagues found that Nivolumab is safe and effective in advanced EC patients who are refractory to standard chemotherapy (28). Jiyun Lee and colleagues found that Nivolumab showed some efficacy as second-line therapy for ESCC in a phase III trial, but the improvement of OS was not significant (29). Through analysis, Qu and colleagues found that overexpression of PD-L1 in ESCC might relate to short OS. However, the difference was not statistical significant (*P*=0.07) (30). Some scholars have also found that the overexpression of PD-L1 in ESCC is related to its disease-free survival (DFS), but it has no correlation with its prognosis (31).

Therefore, the current targeted therapies for EGFR and PD-1 respectively have encountered bottlenecks. The solution is to dig deeper into the molecular mechanisms of ESCC and find other sensitive targets. In the future, combination therapy with multiple molecular-specific targeted drugs may be a good option for the treatment of ESCC.

### Environmental Factors and Probably Genetic Mechanisms of ESCC Development

The causes of ESCC are complex. Environmental and genetic factors are contributors for ESCC formation. It is believed that pathogenic genes have an important influence on it.

Previous studies have found that ESCC has a variety of related environmental predisposing factors (32). Such as tobacco (33, 34), alcoholic beverages (35, 36), little or no-intake vegetables and fruits (37, 38), pickled vegetables (39), hot foods (40) and so on. If the body is exposed to these factors for a long time, it may increase the susceptibility of ESCC. Some studies found a genetic link with these exposures for developing ESCC. Chen Wu and colleagues found that there was a gene-environment interaction between alcohol abuse and genetic variation in alcohol metabolic pathways that leads to the development of ESCC. It has been reported that drinkers who carried both the risk alleles of *ADH1B* and *ALDH2* had the highest risk to develop cancer (41).

In ESCC, Yongmei Song and colleagues found genomic alterations in several important pathways (e.g., the RTK-RAS and AKT pathways) and genes (e.g., *PIK3CA*) (42).

De-Chen Lin and colleagues found that the MAPK and PI3K pathways were activated through a variety of mechanisms in ESCC (16). At the same time, several potentially altered genes have been identified in ESCC. Such as *ERBB*, *HDAC*, *PI3K family*, *XPO1*, *FGFR1*, *TP53*, *JAK-STAT3*, and *mTOR-rpS6K* were defined to be recurrent candidate druggable targets (16). These genes have implications for future molecular studies.

**Figure 2:**
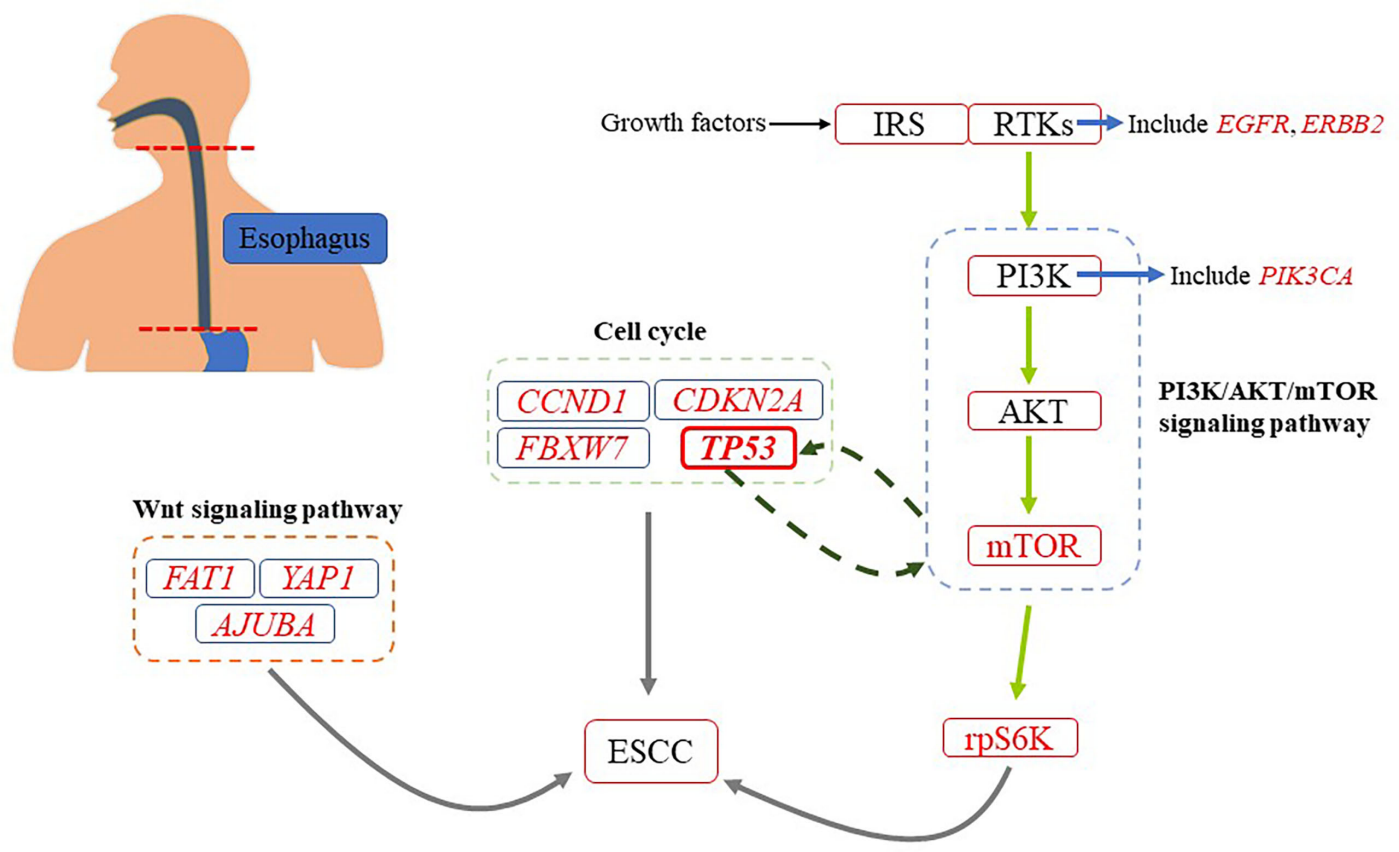
A simplified diagram of genetic changes about ESCC. There are many genetic changes associated with ESCC. It has been found that PI3K/AKT/mTOR pathway is closely related to the occurrence and development of ESCC.

The results of a whole-exome sequencing conducted by Genta Sawada and colleagues are consistent with those of De-Chen Lin and colleagues. Their sequencing included 144 Japanese patients with ESCC, including neoplastic and non-neoplastic esophageal tissues (43). In addition, they also found some other gene mutations in many tumor tissues (43). For example, some mutations in genes that regulate cell cycle (*TP53*, *CCND1*, *CDKN2A*, and *FBXW7*) and epigenetic process (*MLL2*, *EP300*, *CREBBP*, and *TET2*). It should be noted that *TP53* plays a role in inhibiting carcinogenesis in organisms (44). The most common genetic change in a variety of human cancers is the mutation of *TP53* (45). According to reports, the mutation rate of *TP53* in ESCC ranges from 60% to 93.1% (16, 43). Many researchers have found that *TP53* is closely related to PI3K/AKT/mTOR pathway, which may be involved in the occurrence and development of tumors (**Figure 2**). Such as, it has been found that *TP53* can negatively regulate PI3K/AKT/mTOR pathway by upregulating related proteins (45). Besides, it was also found that PI3K/AKT/mTOR pathway could negatively affect the expression of *TP53* by upregulating MDM2, which promotes the degradation of p53 (A protein encoded by *TP53*) (44). Mutations were also found in some key genes of signaling pathways, such as NOTCH, WNT (*FAT1*, *YAP1*, and *AJUBA*) and RTK-PI3K (*PIK3CA*, *EGFR*, and *ERBB2*) (43).

**Figure 3:**
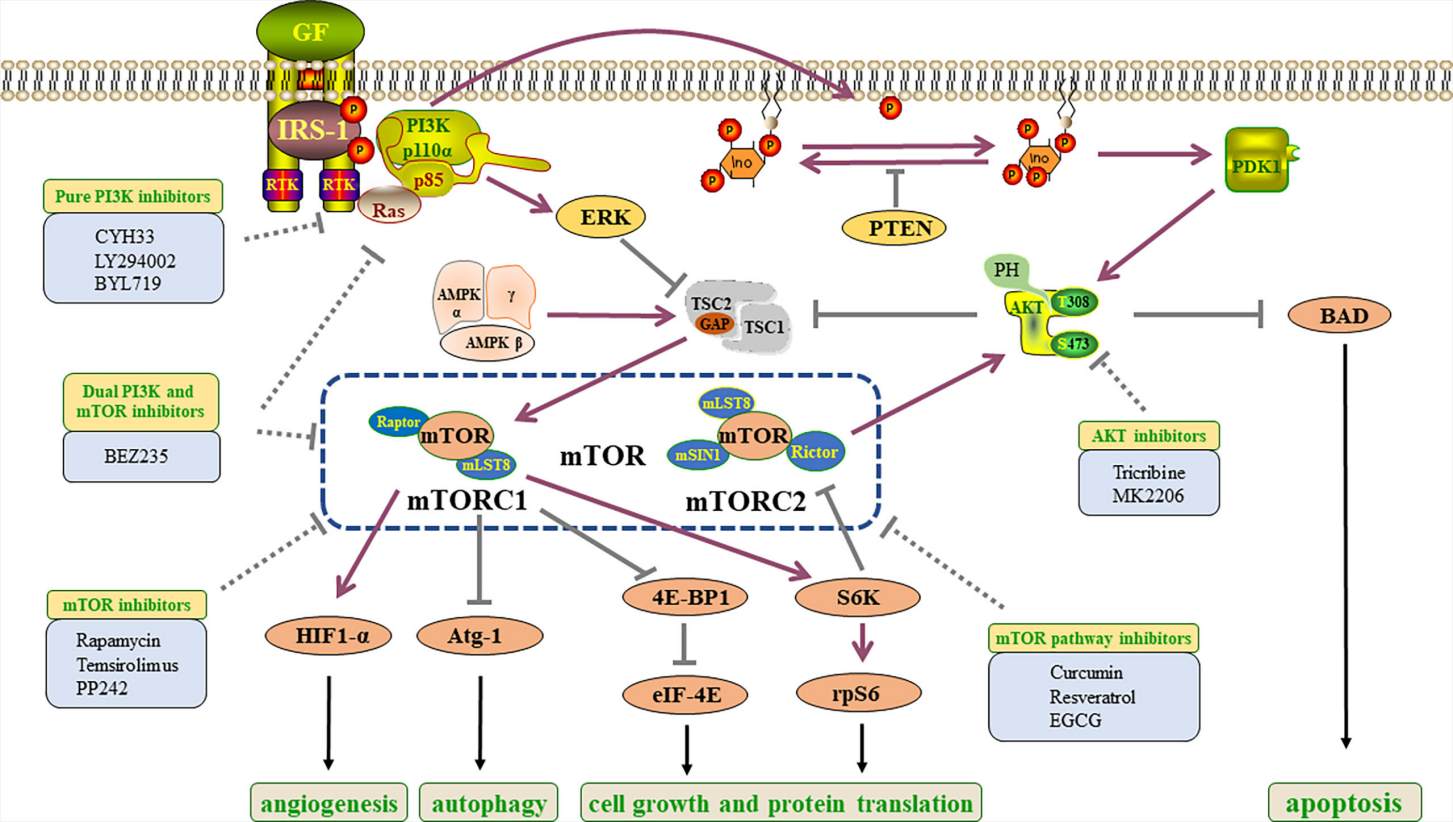
Constituent elements and inhibitors of PI3K/AKT/mTOR pathway in ESCC. Growth factors bind to RTKs to activate the PI3K/AKT/mTOR pathway, which directly and indirectly results in tumorigenesis, the activation of protein translation and angiogenesis, the inhibition of apoptosis and autophagy. GF, growth factors; PI3K, phosphatidylinositol 3-kinase; IRS1, insulin receptor substrate 1; RTK, receptor tyrosine kinase; PIP2, phosphatidylinositol 4,5-bisphosphate; ERK, extracellular signal-related kinase; PIP3, phosphatidylinositol 3,4,5-trisphosphate; TSC, tuberous sclerosis protein; PTEN, phosphatase and tensin homolog; PDK1, pyruvate dehydrogenase lipoamide kinase isozyme 1; AMP,: AMP-activated protein kinase; AKT, protein kinase B; mTORC, mammalian target of rapamycin complex; BAD, Bcl2-related death protein; Raptor, regulatory associated protein of mTOR; HIF1-α, Hypoxia-inducible factor 1-α; mLST8, mammalian lethal with sec-13 protein 8; eIF4E, eukaryotic translation initiation factor 4E; mSIN1, mammalian stress-activated MAPK- interacting protein 1; Rictor, rapamycin insensitive companion of mTOR; Atg-1, autophagy-related protein 1; 4E-BP1, 4E-binding protein 1; S6K, ribosomal S6 kinase; rpS6, ribosomal protein S6.

At present, abnormal activation of PI3K signaling has been found in ESCC, and genetic mutations of *PI3K*, *AKT* and *mTOR-rpS6K* have been found. The researchers found that *EGFR*, *ERBB2* and *FGFR1* genes were mutated, and their downstream key pathways were PI3K/AKT/mTOR pathway (16, 43). To some extent, the functional realization of the three components depends on PI3K/AKT/mTOR pathway. Therefore, the occurrence and development of ESCC are closely related to the PI3K/AKT/mTOR pathway (**Figure 3**).

At the same time, PI3K/AKT/mTOR pathway has been proved to be an important pathway controlling growth and metabolism in cells, which is an important guarantee for the survival and normal function. Abnormal activation of PI3K/AKT/mTOR pathway has been found in many tumors, and inhibition of this pathway has also achieved certain therapeutic effects for tumors.

## PI3K/AKT/mTOR Signaling Pathway

The PI3K/AKT/mTOR signaling pathway plays an important role in basic functions of cell growth, apoptosis, translation, and cell metabolism (46). There were found abnormal expressions of PI3K/AKT/mTOR signaling pathway in many tumors (47). These evidences suggested that this pathway acting as an essential part in the development of tumors and suggested their potential as new therapeutic targets.

PI3Ks constitute an important enzyme family namely the lipid kinase family. It can be divided into three categories. The class I PI3Ks are heterodimers composed of catalytic subunits and regulatory subunits (48). Class I PI3Ks could be subdivided into class IA and IB enzymes. The class IA consists of three catalytic subunits (p110α, p110β and p110δ) encoded by *PIK3CA*, *PIK3CB* and *PIK3CD* genes, which can be activated by receptor tyrosine kinases (RTKs). While the class IB is composed of p110γ (a catalytic subunit) encoded by *PIK3CG* and activated by G protein-coupled receptors (GPCRs) (49, 50). The regulatory subunits of class IA and IB are also different in structure (51–54). Class II PI3Ks consist of three different subtypes (PI3K-C2α, PI3K-C2β and PI3K-C2γ) (55). Class III PI3Ks are composed of two subunits (Vps34 and Vps15), and could play an important role in the autophagy and phagocytosis pathway of lysosomes (56).

Class I PI3Ks are the research hotspot of PI3K signal transduction. In addition, class IA PI3Ks are widely found in carcinogenic processes. RTK or GPCR activation enrolls class I PI3Ks into the plasma membrane, where p85 (regulatory subunit) -mediated inhibition of p110 is released and p110 directly phosphorylates PIP2 (phosphatidylinositol 4,5-bisphosphate) into PIP3 (phosphatidylinositol 3,4,5-triphosphate) (57). This lipid is similar to the model of second messenger, which activates downstream proteins and participates in cell growth and survival (58). Phosphatase and tensin homolog (PTEN) can dephosphorylate the third site of the PIP3 inositol ring, result in the conversion to PIP2. It is a negative regulatory factor that inhibits the transduction of PI3K signal to pyruvate dehydrogenase kinase 1 (PDK1) (59) (**Figure 3**).

AKT is a serine/threonine kinase and a key downstream signal of PI3K (60). AKT has three subtypes: AKT1, AKT2 and AKT3. Overexpression and phosphorylation of AKT can be found in a variety of cancers (61). Sundaramoorthy Revathidevi and colleagues searched the TCGA data and found that compared with other activation methods such as amplification, overexpression and phosphorylation, the activation of AKT by mutation was rare (60). In many cancers, methylation of its upstream regulators, including PTEN, has been shown to activate AKT (62). Also the activation mutation of *PI3K*, *RAS* can potentially activate *AKT* (63). It is established that PI3K can directly activate mTORC2, and the activated mTORC2 can activate AKT (59) (**Figure 3**).

mTOR signal is one of the key genetic variation targets in cancers, which is often associated with tumor occurrence and progression. The mTOR protein is a serine-threonine kinase of PI3K related family, which is a part of mTORC1 and mTORC2 complexes. These two complexes have different structures and functions (64). The mTORC1 contains regulatory associated protein of mTOR (Raptor), while the mTORC2 contains rapamycin insensitive companion of mTOR (Rictor) (64). Which can explain that mTORC1 is sensitive to rapamycin while mTORC2 is not sensitive to rapamycin treatment. The two both contain mammalian lethal sec-13 protein 8 (mLST8). In addition, mTORC2 contains mammalian stress-activated MAPK-interacting protein 1 (mSIN1; also known as MAPKAP1) (64) (**Figure 3**). The tuberous sclerosis complex (TSC) is a factor that can regulate mTORC1, and it is also one of the convergence points of multiple pathways *in vivo*. TSC is a heterotrimer consisted of TSC1, TSC2 and TBC1D7. At the same time, TSC functions as a GTPase activation protein (GAP) could activate the ras homolog enriched in brain (RHEB), a small GTP enzyme. When AKT is activated, it can mediate TSC2 phosphorylation, inhibit TSC1/2 complex and activate mTORC1 signal (65).

Growth factors, amino acids, and oxygen can activate mTORC1. When it was activated, it can participate in protein, lipid and nucleotide synthesis and autophagy (59). For example, after mTORC1 activation, the phosphorylation of its downstream signal molecule 4E-binding protein 1 (4E-BP1) could be inhibited, thus the eukaryotic translation initiation factor 4E (eIF4E) will be released to participate in protein synthesis (59). Autophagy-related protein 1(Atg-1) is a node in several different signaling pathways regulating autophagy *in vivo*. mTORC1 is one of the upstream signals of Atg-1, and the activation of mTOR signal can inhibit the autophagy induction ability of Atg-1 (66) (**Figure 3**).

mTORC2 is often over-activated in cancer cells, and can promote cell survival and migration through phosphorylation of Akt Ser 47 (67, 68). In addition, mTORC2 can regulate additional physiological functions by phosphorylating different substrates such as glycolytic enzyme pyruvate dehydrogenase kinase 1 (PDHK1), serum and glucocorticoid induced kinase (SGK), protein kinase C ζ (PKC ζ) and so on (59, 69, 70).

### The Influence of PI3K/AKT/mTOR Pathway on ESCC Development

Many studies have found that the PI3K/AKT/mTOR pathway is associated with cell proliferation, apoptosis, autophagy, and drug resistance of ESCC. Therefore, therapy targeting PI3K/AKT/mTOR pathway should be a promising therapeutic strategy.

### Influence on Cell Proliferation and Apoptosis

The proliferation is inextricably linked with apoptosis, whether in normal cells or in tumor cells (71). Numerous studies have found that mTOR regulates cell growth and division. mTORC1 directly activates the ribosomal protein S6 kinase (p70S6K) and inhibits 4E-BP1, thereby increasing translation. At the same time, to a certain extent, it can regulate cell proliferation by controlling cell cycle. And mTORC2 promotes metabolism mainly by activating AKT2.

Shau-Hsuan Li and colleagues demonstrated overexpression of phosphorylated mTOR, p70S6K, and 4EBP1 in 56% tumor tissues of ESCC patients (72). Survival analysis also found that p-mTOR and p-p70S6K overexpression, Ki-67 index >50% were associated with poor OS. Among them, the overexpression of p-p70S6K can be considered as an independent prognostic indicator of ESCC. It is confirmed that everolimus (an inhibitor of mTOR) can inhibit the growth of ESCC in both cell lines and transplanted tumor models (72). Before this, Guiqin Hou and colleagues found that rapamycin and siRNA against mTOR can rapidly inhibit the expression of mTOR and phosphorylation of p70S6K and 4EBP1. In addition, inhibition of mTOR can also make cell cycle arrest at G0/G1 phase and induce apoptosis of ESCC cells (73).

In an experiment conducted by Jiarui Yu and colleagues, these scientists explored the effects of Gambogic acid (GA), the mainly active component secreted by Garcinia hanburryi tree, on the ESCC cells (74, 75). It was found that GA could inhibit the proliferation, migration, and invasion of ESCC cells. Meanwhile, GA induced dose-dependent apoptosis in ESCC cells by inhibiting the expression of Bcl2 and up-regulating the expression of apoptosis-related proteins such as Bax and cleaved-caspase3/9 (75). The probably mechanism was that GA can down-regulate the levels of PI3K, p-AKT and p-mTOR, and promote the expression of PTEN in ESCC cells (75).

These experimental data suggest that proliferation and apoptosis are closely related to PI3K/AKT/mTOR pathway in ESCC.

### Influence on Cell Autophagy

Autophagy restricts malignant transformation, balances cell metabolism, and maintains cell survival, but autophagy can promote the cells growth and progression of the cancer (66). Many studies have confirmed that autophagy can protect the cancer cells from anticancer therapy by blocking the apoptotic pathway (also called protective autophagy), to keep the cancer cells alive, allowing them to grow and metastasize (76). Some studies have confirmed that autophagy is mainly induced through PI3K/AKT/mTOR signaling pathway (77). If this pathway is blocked, the autophagy will be inhibited, and the apoptosis will be activated. They together enhance the sensitivity of tumor cells to treatment (77). Beclin-1 synergistic with PI3K pathway enhances autophagy vacuole and activates autophagy cascade reaction (78). Microtubule-associated protein light chain 3 (LC3), now widely used as a monitoring autophagy body formed by specific molecular markers (79). Yu and colleagues found that enhanced autophagy was associated with cisplatin resistance in ESCC cell lines (80). O’Donovan and colleagues found that in drug-resistant ESCC cells, LC3-II levels were significantly increased after treatment with 5-fluorouracil (81). However, inhibition of autophagy induction by siRNA targeting Beclin1 and ATG7 significantly enhanced the effect of 5-fluorouracil (81). These studies suggest that in ESCC cells, autophagy acts as a protective mechanism to promote cell survival during antitumor therapy, leading to therapeutic resistance. In addition, Le Yu and colleagues found that autophagy inhibition can enhance the sensitivity of ESCC cells to cisplatin *in vivo (* 80). Chi Lu and colleagues reported that ionizing radiation activates autophagy in ESCC cell lines. In addition, they found that inhibiting autophagy can enhance apoptosis and cell cycle arrest *in vitro* induced by radiation (82). Yan Cai and colleagues found that chloroquine inhibited the growth and proliferation of ESCC cell EC109, and this was mediated by regulating autophagy (83).

## The Related Inhibitors to PI3K/AKT/mTOR Pathway of ESCC

### Pure PI3K Inhibitors

According to selectivity, pure PI3K inhibitors can be divided into two types: pan PI3K inhibitors and selective isoform PI3K inhibitors (84). CYH33 is a novel selective inhibitor of PI3Kα with a unique structure. Jia-jie Shi and colleagues reported that CYH33 combined with radiotherapy can synergistically inhibit the proliferation of ESCC (85). Clinical trial of CYH33 in the treatment of advanced ESCC(NCT03544905) is currently under way (**Table 1**). LY294002 was identified as a generic PI3K inhibitor. Guiqin Hou and colleagues found that LY294002 could inhibit proliferation of ESCC cells through PI3K/AKT/mTOR/p70S6K signaling pathway. However, LY294002 triggered AKT (Ser473)/PRAS40 (Thr246) feedback activation mediated by mTORC2 in Eca109 and Ec9706 cells. This may lead to limited therapeutic effect of LY294002 on ESCC (86). Therefore, the role of a single PI3K inhibitor is limited. Further experiments by Guiqin Hou and colleagues highlighted that shRNA inhibition of Rictor could reduce phosphorylation of AKTSer473 and Thr308 sites, and counteract activation of AKT (Ser473)/PRAS40 (Thr246) induced by LY294002, which significantly improved the sensitivity of ESCC cells to LY294002 *in vitro* and *in vivo (* 86). BYL719 is a PI3Kα inhibitor. Moshe Elkabets and colleagues found that AXL is involved in ESCC resistance to BYL719 (87). The mechanism of drug resistance may be that AP-1 transcription factors c-JUN and c-FOS regulate the overexpression of AXL (88). The combination of BYL719-SP600125 (blocking JNK signaling pathway) has achieved certain results *in vitro* and *in vivo (* 88). For PI3K inhibitor, the application of PI3K inhibitor combined with other drugs should be a hot spot in the future. However, the result of a completed clinical trial of combined drug use was not satisfactory. Combined application of LJM716(HER3 targeting antibody) and BYL719 (NCT01822613) in ESCC patients, the tumor did not shrink as expected.

**Table 1:** The researches about inhibitors of PI3K/AKT/mTOR pathway in ESCC.

Classification	Drug	Target	Administration	Latest researches in ESCC	Trial Number
PI3K inhibitors	Rigosertib	PI3K	Oral, parenteral	Clinical trials	NCT01807546
	LY294002	PI3K	Suggest not to use in clinical	Pre-clinical	/
	BYL719	PI3Kα	Oral	Clinical trials	NCT01822613
	CYH33	PI3Kα	Oral	Clinical trials	NCT03544905
AKT inhibitors	MK2206	AKT	Oral	Pre-clinical	/
	Tricribine	AKT	Parenteral	Pre-clinical	/
mTOR inhibitors	Rapamycin	mTORC1	Oral	Pre-clinical*	/
	Temsirolimus	mTORC1	Parenteral	Pre-clinical*	/
	PP242	mTORC1/2	Parenteral	Pre-clinical	/
Dual PI3K and mTOR inhibitors	BEZ235	PI3K, mTORC1/2	Oral	Pre-clinical	/
mTOR pathway inhibitors	Curcumin	mTOR pathway	Oral	Pre-clinical	/
	Resveratrol	mTOR pathway	Oral	Pre-clinical	/
	EGCG	AKT, ERK1/2, mTOR pathway	Oral	Clinical trials	NCT05039983

*FDA approved.

### AKT Inhibitors

Few Akt inhibitors are currently used in clinical trials. Tricribine (TCN), an Akt inhibitor, significantly inhibited p-Akt, HIF-1α, and VEGF expression *in vitro* and *in vivo*, enhancing the radiosensitivity of ESCC *in vitro* and *in vivo (* 89). ESCC has been proved to have a close relationship with PI3K/AKT/mTOR. The phosphorylation at Thr308 and at Ser473 is both necessary for full AKT activation. MK-2206 is an oral inhibitor targeting on all three AKT subtypes. Ni Shi and colleagues found that in ESCC cells, the phosphorylation level of AKT at Ser473 only slightly decreased upon treatment with MK2206 (**Table 1**) (90). The effect of MK2206 alone in the mouse model of ESCC was also not ideal. However, in a clinical trial conducted by Timothy A. Yap and colleagues, it was found that MK-2206 has a certain therapeutic effect on solid tumors such as lung and colorectal cancer (91). The underlying molecular changes in ESCC may be more complex. For example, when MK2206 is used alone, the up or downstream targets of AKT may be activated to affect drug action. Subsequent experiments by Nishi and colleagues found that MK2206 combined with BEZ235 (a co-inhibitor of PI3K and mTOR) enhanced the inhibiting of proliferation in ESCC cells, both *in vivo* and *in vitro*.

### mTOR Inhibitors

The mTOR inhibitors are divided into three generations (92). Current researches are mainly focused on the first and second generations.

#### The First Generation of mTOR Inhibitors

Rapamycin and its analogues are the first generation of mTOR inhibitors. Through allosteric mechanism, they can partially inhibit the activity of mTORC1 and slow down the proliferation of cancer cells (93). Guiqin Hou and colleagues reported that rapamycin could induce apoptosis in ESCC cells. In addition, rapamycin was found to inhibit tumor growth in human ESCC cell line EC9706 in nude mice. Its inhibitory effect was stronger than that of cisplatin used alone. But the combination of rapamycin and cisplatin was the strongest (94). Temsirolimus (CCI-779, TriceITM) is one of these analogues. Toshio Nishikawa and colleagues found that in some ESCC cell lines (such as TE-1, TE-8, and TE-10), the level of mTOR phosphorylation was increased, accompanied by the upregulation of hypoxia-inducible factor-1α (HIF-1α). Temsirolimus significantly inhibited the activation of mTOR and its downstream effector proteins, resulting in decreased proliferation of ESCC cells. Finally, *in vitro*, temsirolimus significantly reduced the size of subcutaneous tumors in nude mice and effectively extended the survival of mice with esophageal carcinoma *in situ* (the cell used for this experiment was TE-8) (95).

At the same time, the first generation of mTOR inhibitors only had little effect on the phosphorylation of 4E-BP1 (96).These inhibitors do not inhibit the activity of mTORC2, so the direct activation of AKT by mTORC2 is not affected. And the negative feedback loop formed after suppressing mTORC1 can activate the PI3K/AKT signal (93). This may be the cause of inhibitor resistance. Now some studies have found that some tumors are resistant to these inhibitors. For example, T Fujishita and colleagues found that everolimus (a rapamycin analogue) had little effect on blocking tumor invasion when used in the later phase of locally aggressive intestinal adenocarcinoma (cis-Apc/Smad4 mice model). But inhibiting mTOR and EGFR or MEK at the same time may be more effective in treating colon cancer (97). As such, the molecular in tumors are more complex than expected, and combination of drugs seems to be a more meaningful route.

Clinical trials with rapamycin and its analogues related to ESCC have not been carried out. Still some researchers have also had limited success in treating a small number of rare cancers with monotherapy, including mantle cell lymphoma and pancreatic neuroendocrine tumors (98, 99).

#### The Second Generation of mTOR Inhibitors

The second generation mTOR inhibitors are some small molecule ATP competitive inhibitors. They can target mTOR or both mTOR and PI3K. Several categories according to the chemical structure exist (92). The pyrazolopyrimidine class is one of them. PP242 is a typical example of a pyrazolopyrimidine. The inhibitory effect of PP242 was stronger than that of rapamycin, and PP242 could inhibit the activities of mTORC1 and mTORC2.

Yu Huang and colleagues examined the antitumor effect of PP242 in ESCC cell lines include Eca-109 and TE-1 (100). As expected, they found that PP242 can weaken the activities of both mTORC1 and mTORC2 signaling in ESCC, stronger than rapamycin. PP242 could inhibit 4E-BP1 phosphorylation and abrogate PI3K/AKT feedback activation relying on mTORC1 (100). It seems that the anti-tumor effect of the second generation of inhibitors should be far more obvious than that of the first generation. In ESCC cells, Yu Huang and colleagues found PP242 can effectively inhibit the proliferation, induce apoptosis, and arrest cell cycle (100).

PP242 is not tested in currently ongoing clinical trials. TAK-228 (derived from PP242) was well tolerated as a single agent and showed initial therapeutic activity in hematological malignancies (NCT01118689) (101).

### Dual PI3K and mTOR Inhibitors

Regarding second generation mTOR inhibitors, some compounds were found to target both PI3K and mTOR (92). BEZ235 is one of them. Ning Wu and colleagues found the activity of p-AKT, p-mTOR, and p-p70S6K can be reduced significantly by BEZ235 in ESCC cells include Eca-109 and TE-1. This inhibitory effect can induce autophagy and apoptosis of human ESCC cells (102). At the same time, they found that BEZ235 combined with Trichostatin A(histone deacetylase inhibitor) had better tumor inhibition effect than single drug (102).Clinical trials of dual PI3K and mTOR inhibitors have not been conducted in the ESCC. But the clinical trials of BEZ235 in other tumors are ongoing. In a clinical trial, the dual PI3K and mTOR inhibitor, PF-05212384 was found to have manageable safety and antitumor activity. This trial provides support for further clinical studies in patients with advanced solid malignancies (NCT 00940498) (103).

### mTOR Pathway Inhibitors

Some natural polyphenols extracted from plants, such as curcumin and resveratrol, have been confirmed to inhibit mTOR signaling pathway directly or indirectly in certain tumors (92). Researchers have also explored the role of these extracts in ESCC (104, 105).

Curcumin is a polyphenolic compound extracted from turmeric roots. It is safe, non-toxic, and has anti-tumor effects in the human body (106). Many studies have shown that curcumin and PI3K/AKT/mTOR signaling pathway are closely related. Lian Deng and colleagues found that curcumin combined with docetaxel can induce apoptosis and autophagy in ESCC cells, which may be based on the PI3K/AKT/mTOR signaling pathway (104).

Resveratrol is rich in grapes, red wine, and peanuts (107). It is a plant defensin that has specific cytotoxicity for multiple carcinoma cells (such as melanoma and breast cancer), with certain treatment potential (108, 109). Qishan Tang and colleagues has confirmed that resveratrol can induce cell cycle arrest at the sub-G1 phase and result in subsequent apoptosis, in a dose-dependent manner (110). They also confirmed that resveratrol can inactivate the mTOR signal (110).

Epigallocatechin-3-gallate (EGCG), a primary tea polyphenol, has been shown to inhibit the growth of certain human cancer cells (111). The mechanisms of inhibiting tumor are antioxidation, inhibiting cell proliferation and angiogenesis, as well as increasing cancer apoptosis (111). Yao-Kuang Wang and colleagues has confirmed that EGCG can inhibit the proliferation and colony formation of arecoline-induced ESCC cells by inhibiting AKT and ERK1/2 pathway (105). Exactly, an important downstream signal of AKT and ERK1/2 is mTOR. A clinical trial on EGCG (NCT05039983) in ESCC is currently ongoing in China.

These natural compounds can inhibit the growth of ESCC cells and are inseparable from mTOR signal.

## eIFs and PI3K/AKT/mTOR Pathway

### PI3K/AKT/mTOR Pathway Can Regulate eIFs to Influence the Translation

Translation is an important and complicated process of gene expression in eukaryotes. Translation mainly includes four processes: initiation, elongation, termination, and ribosome recycling (112). The regulation of translation mainly takes place at initiation phase and which is the rate limiting phase of protein synthesis (113). The regulators of translation initiation are the eIFs. The activation of RTKs, MAPK and PI3K/AKT signaling pathways could be stimulated by some signals which promoting tumorigenesis (114). These pathways play an important role in the regulating of eIF functions (115). Both MAPK and PI3K/AKT pathways regulate the functions of eIFs *via* mTOR. Therefore, mTOR plays a leading role in the regulation of eIF functions (115, 116). Their mis-regulation usually causes abnormal translation, synthesizes aberrant proteins, finally leading to tumorigenesis (117–120).

### Overview on the Role of eIFs in Translation Initiation

The initiation process of translation begins with the formation of the 43S pre-initiation complex (121). The 43S initiation complex consists of 40S ribosomal subunit, eIF2–GTP–Met-tRNAiMet, eIF1, eIF1a and eIF3 (121, 122). The 43S initiation complex can then be guided by the eIF4F complex to bind with mRNA. The eIF4F complex is a heterotrimer composed of eIF4A, eIF4E and eIF4G subunits (123). The mRNA was scanned by 43S initiation complex. With the assistance of eIF1, the tRNA anticodon ring correctly binds to the start codon AUG on the mRNA (121). eIF4A has helicase activity. eIF4E binds to the mRNA’s cap structure. It is usually bonded with 4E-BP1. Once 4E-BP1 was phosphorylated, the eIF4E could be released. The role of eIF4G is to link mRNA with ribosomes (123, 124). eIF3 acts more like a scaffold, links other eIFs with 40S ribosomal subunit (125). When the complex encounters the correct AUG start codon, eIF2 will be hydrolyzed, and other eIFs will be released. At the last stage of translation initiation, eIF2 is in an inactive GDP binding state, the GTP bound to eIF5B is hydrolyzed, and these translation factors are separated from the ribosome (122, 126). In addition, eIF6, which has not yet been mentioned, is the first eIF associated with the 60S subunit that modulates translation in response to extracellular signals (127, 128).

### eIFs-Potential Therapeutic Targets in Tumors

Moreover, some researchers found that changes in the expression of certain translation promoters (primarily increased expression) were associated with the development of specific tumors (129–131). For example, eIF1A is a small 17kDa promoter and highly conserved in all eukaryotes. Somatic mutations in the N-terminal tail (NTT) of eIF1A have been found to be associated with uveal melanoma, thyroid cancer, and ovarian cancer (132–134). Urmila Sehrawat and colleagues found that eIF1A could regulate different mRNAs differently in mammalian cells (135). The eIF1A NTT mutants enhanced the scanning of the 5 ‘UTR-containing cell cycle genes, possibly affecting the cell cycle and promoting cell proliferation (135). eIF3H is one of the central subunits of eIF3 complex. It has been observed that eIF3H is often amplified in breast and prostate cancer together with proto-oncogene Myc (136, 137). Researchers have found the amplification of eIF3H gene in colorectal cancer and non-small cell lung cancer (NSCLC) through genome-wide analyses and fluorescent *in situ* hybridization (FISH) (117, 138, 139). In addition, the expression level of eIF3H is positively correlated with the poor differentiation and invasive growth of prostate cancer (117, 137).

In ESCC, there are also some limited studies on eIFs. For example, Ting Liu and colleagues found that eIF4E increased significantly in clinical ESCC tissues and ESCC cell lines, and its expression level was associated with lymph node metastasis, TNM period, and ESCC’s overall and disease-free survival (140). After using the shRNA knockout eIF4E, it was found that the induced cytotoxicity by cisplatin has increased in the ESCC cell lines, and the chemosensitivity to cisplatin in xenograft tumor models also has increased (140). Hong Yang and colleagues found that excessive expression of eIF5A2 in ESCC cells resulted in increased chemoresistance to 5-fluorouracil, docetaxel, and taxol. Conversely, the shRNAs of eIF5A2 could increase the sensitivity of tumors to these chemotherapeutic drugs. It was found that in patients who underwent taxane-based chemotherapy after esophagectomy, eIF5A2 overexpression was associated with poor total survival rate (*P <*0.05) *(* 141). Therefore, targeting eIF4E or eIF5A2 may be a feasible method of improving ESCC chemotherapy sensitivity. However, eIFs in ESCC still require more researches.

These previous examples reveal eIFs may be a promising target for future tumor treatment. At present, there are some inhibitors of eIFs in preclinical and clinical trials (**Table 2**). Ribavirin is an antiviral drug approved by the Food and Drug Administration (FDA) for hepatitis C, which can also treat syncytial virus infection and viral hemorrhagic fever (144–146). Importantly, ribavirin has been extensively documented as an inhibitor of eIF4E (147–149).

**Table 2:** The inhibitors of eIFs in tumor and the specific tumor types that can be inhibited in clinical trials.

Classification	Target	Administration	Development	Tumor type	Trial Number
4EGI-1	elF4F	Not published	Pre-clinical	/	/
Ribavirin	elF4E	Oral, parenteral	Clinical trials*	Acute Myeloid Leukemia (142)	NCT01056523
ISIS 183750	elF4E	Parenteral	Clinical trials	Colorectal Cancer (143)	NCT01675128
LY2275796	elF4E	Parenteral	Clinical trials	/	/
eFT226	eIF4A1	Parenteral	Clinical trials	/	/

*FDA approved.

JingJin and colleagues found an overexpression of eIF4E in most ovarian cancer patients. In addition, the eIF4E function is critical for the growth and survival of tumors (150). eIF4E inhibition was found to be achieved at clinically achievable doses of ribavirin. Inhibition of eIF4E by ribavirin may be a potential therapeutic approach to improve clinical management of ovarian cancer (150). Sakibul Huq and colleagues found that ribavirin enhanced radiosensitivity in nasopharyngeal carcinoma (NPC). At the same time, it can inhibit the expression of various proteins which are all overexpressed in NPC and correlated with poor prognosis, also can inhibit the mTOR/eIF4E axis (151). These studies indicate that ribavirin is a potential targeted drug for tumor therapy.

Several clinical trials related to ribavirin are underway, such as oropharynx squamous cell cancer (NCT01721525), acute myeloid leukemia (NCT01056523), melanoma (NCT00897312).

## Conclusions and Discussions

ESCC is a complex disease, the external predisposing factors and genetic mutations both have an important impact on oncogenesis and tumor progression. So, we should focus on the prevention, warning high-risk individuals away from alcohol, cigarettes, and so on. At the same time, we should pay attention to the screening of disease and improve the early diagnostic rate. Once ESCC patients could be diagnosed in the early stage, their prognosis and living quality will be improved significantly.

From the global cancer report, the incidence and mortality rate of ESCC are currently ranked tenth and sixth (1). This data shows that the current situation of ESCC is not optimistic. At present, neoadjuvant chemoradiotherapy and esophagectomy are the mainstream treatment methods of ESCC. Many researchers focus on treating ESCC by targeted therapy (EGFR or PD-1). There are currently a variety of related drugs used for clinical, and some ESCC patients respond particularly well to them. Still the increase of patients’ overall five-year survival rate has no statistical significance (18, 31).

In all results of ESCC gene testing conducted by several research groups, the abnormal expression of PI3K/AKT/mTOR and its related pathways have been found. In this article, we discussed how the PI3K/AKT/mTOR pathway affects the growth, proliferation, and autophagy of ESCC. It is also discussed that inhibitors in different parts of PI3K/AKT/mTOR pathway can affect the growth and biological behavior of ESCC (**Table 1**). Additionally, the eIFs regulated by PI3K/AKT/mTOR pathway, also has an important influence on the occurrence and development of tumors. Through the discussion, it was found that the PI3K/AKT/mTOR pathway and eIFs could be the future therapeutic target of ESCC.

Still, it should not be ignored that there are only few relevant studies on the application of PI3K/AKT/mTOR pathway inhibitors in ESCC. Most studies have used a single inhibitor and with limited efficacy. At present, there are few studies on the combination of multiple inhibitors with different targets in ESCC ongoing. It may be that researchers have not yet paid attention to the potential therapeutic effects of PI3K/AKT/mTOR pathway and its regulated eIFs in ESCC. It also could be that new drugs are being developed slowly and in fewer varieties. But why the effectiveness of a single inhibitor is limited?

The researchers found that the activation of multiple pathways is the main cause of drug resistance in tumor cells (115). Such as, Jessie Villanueva and colleagues found that melanoma cells show enhanced activation of PI3K signaling after treatment with BRAF inhibitors, leading to drug resistance of tumor cells (152). In ESCC, some researchers found the resistance of tumor cells to EGFR-targeted therapy might be related to the abnormal activation of PI3K/AKT/mTOR pathway (19). Thus, the growth of tumors carrying oncogenes that activate multiple pathways does not depend on a single signaling pathway.

The PI3K/AKT/mTOR pathway and its regulated eIFs have been proved to be a key pathway involved in growth. And many other pathways in the body are inseparable from it. Therefore, the future treatment of ESCC must be related tightly with the PI3K/AKT/mTOR pathway and its regulated eIFs. Simultaneous suppression of multiple targets of this pathway may be a future research focus. One hypothesis: it may be focused more on the co-inhibition of PI3K/AKT/mTOR pathway and eIFs.

In this context, multiple components of several oncogenic signaling pathways and eIFs participated in mRNA translation have been identified as biomarkers with potential diagnostic, therapeutic and prognostic value. Therefore, anti-tumor agents targeting the core elements of protein synthesis and related signaling pathways can get over intratumor heterogeneity and represent as novel promising anticancer drugs.

## Author Contributions

Conceptualization, RH, JH, and YD. Writing—original draft preparation, RH, QD, RY, and QZ. Writing—review, revising and editing, RH, JH, and ZY. All authors have read and agreed to the published version of the manuscript.

## Funding

The project of Department of Science and Technology, Sichuan province, Grant/Award, Number: 2018JY0398.

## Conflict of Interest

The authors declare that the research was conducted in the absence of any commercial or financial relationships that could be construed as a potential conflict of interest.

## Publisher’s Note

All claims expressed in this article are solely those of the authors and do not necessarily represent those of their affiliated organizations, or those of the publisher, the editors and the reviewers. Any product that may be evaluated in this article, or claim that may be made by its manufacturer, is not guaranteed or endorsed by the publisher.
